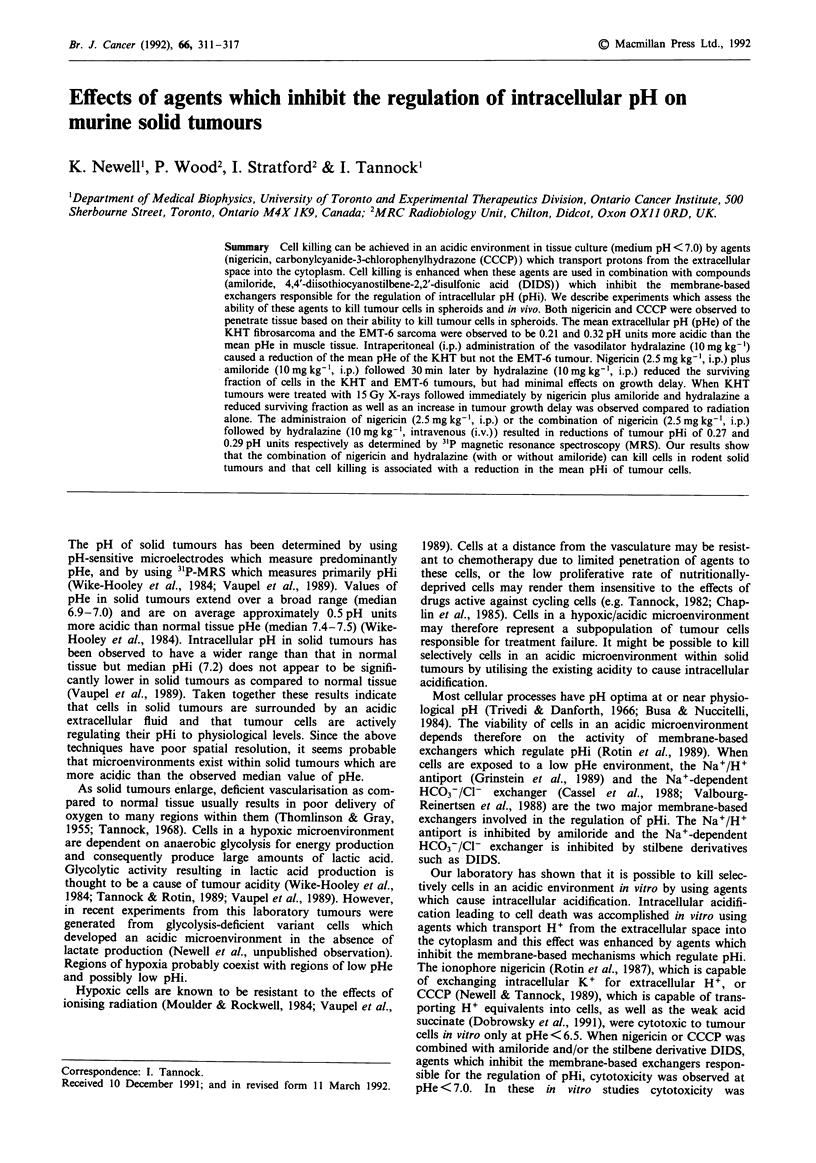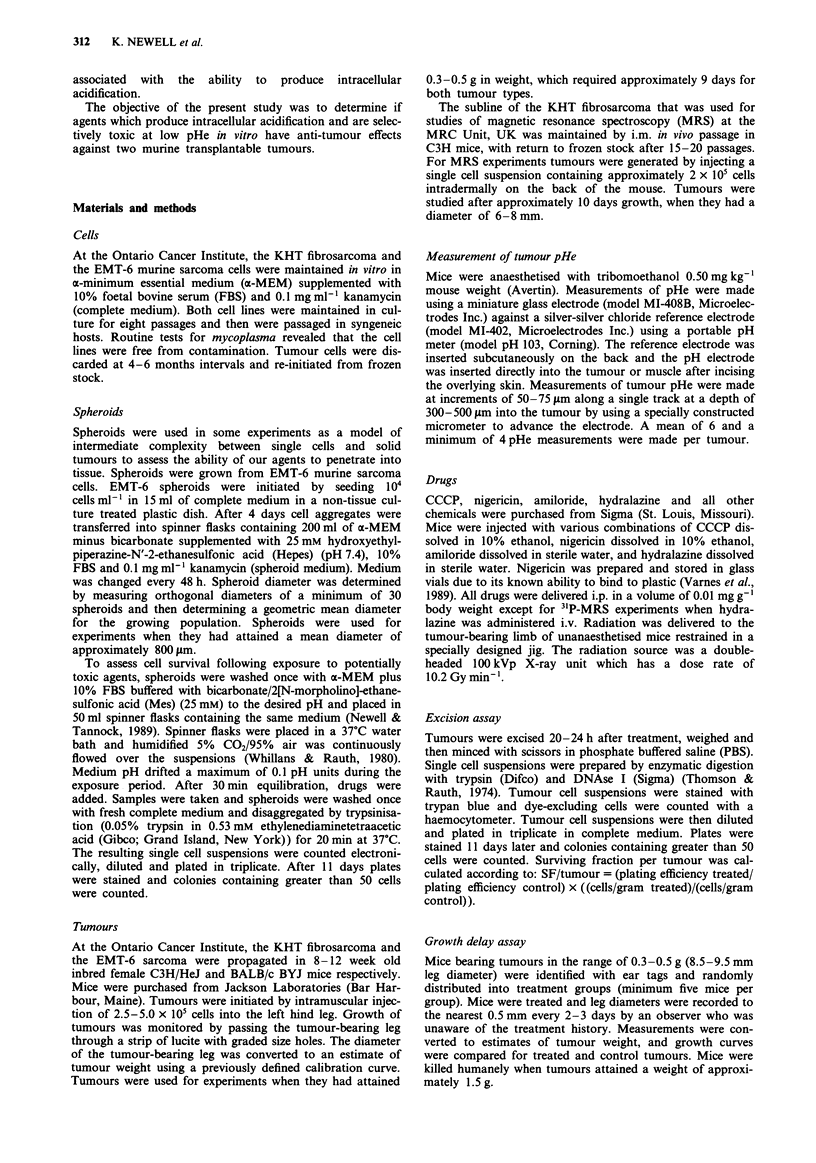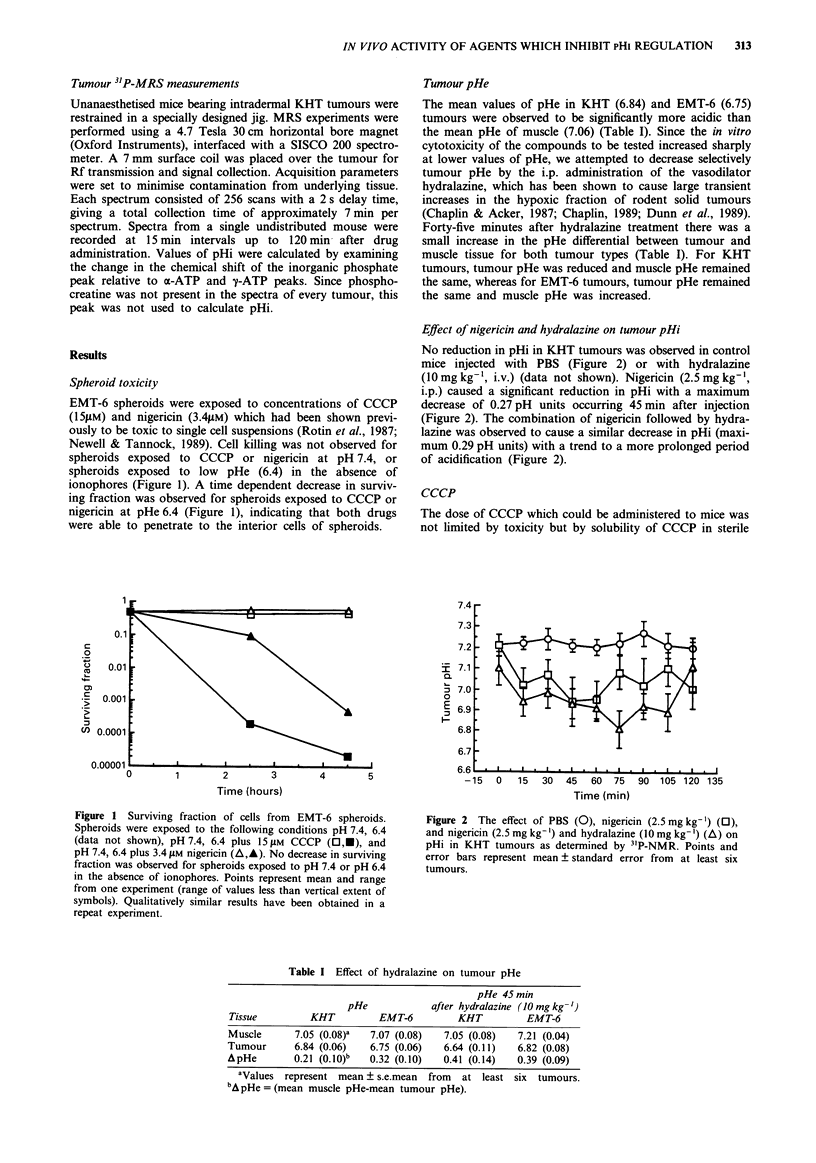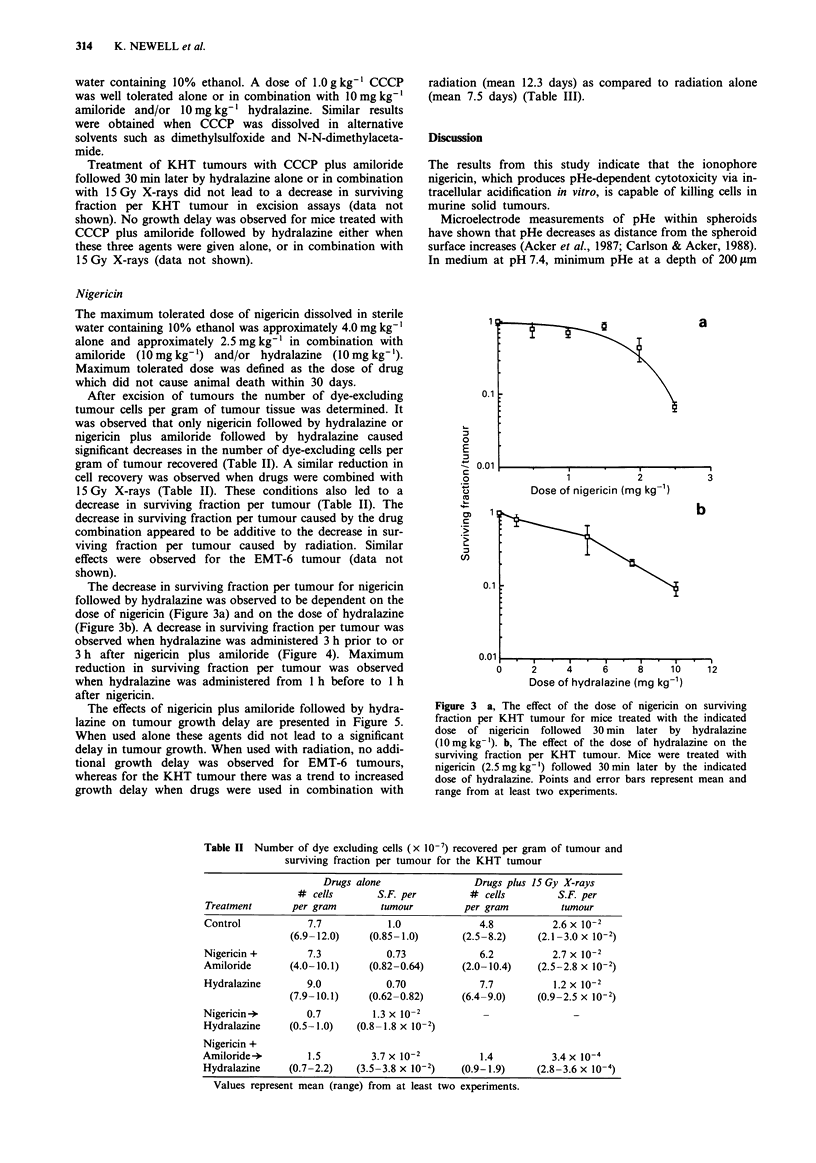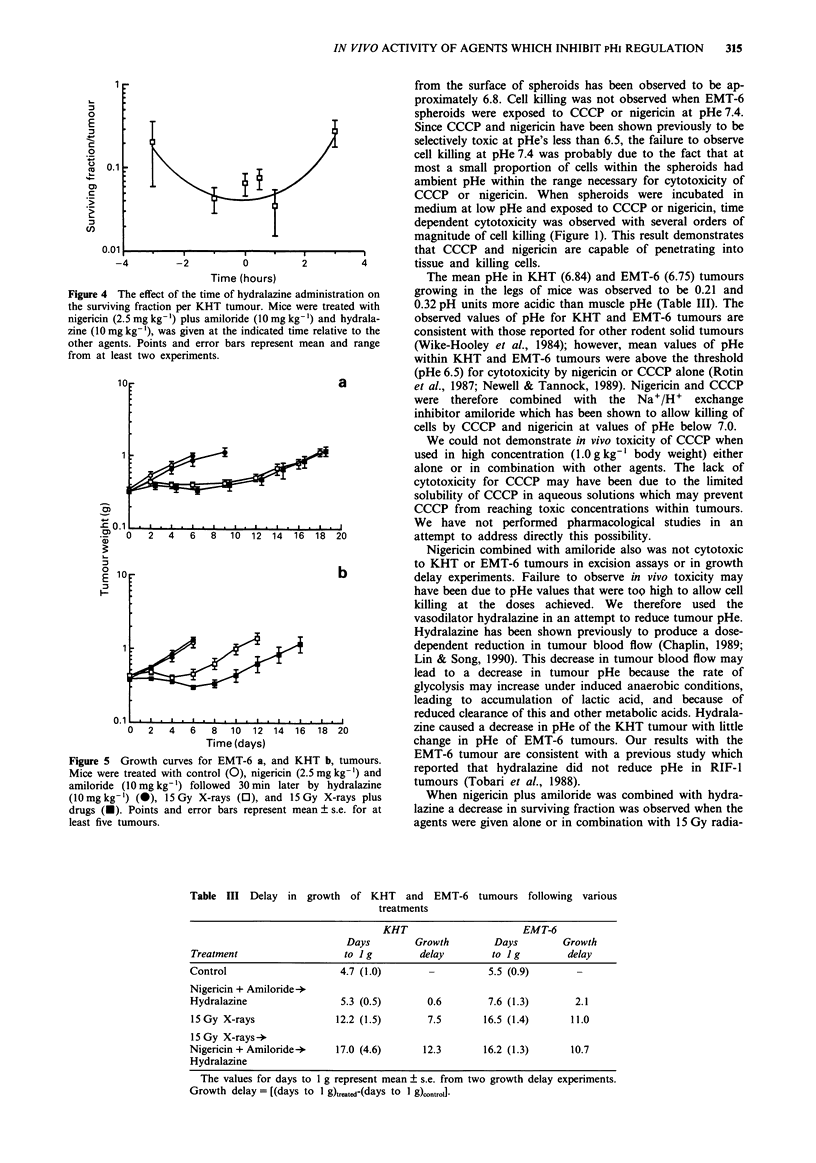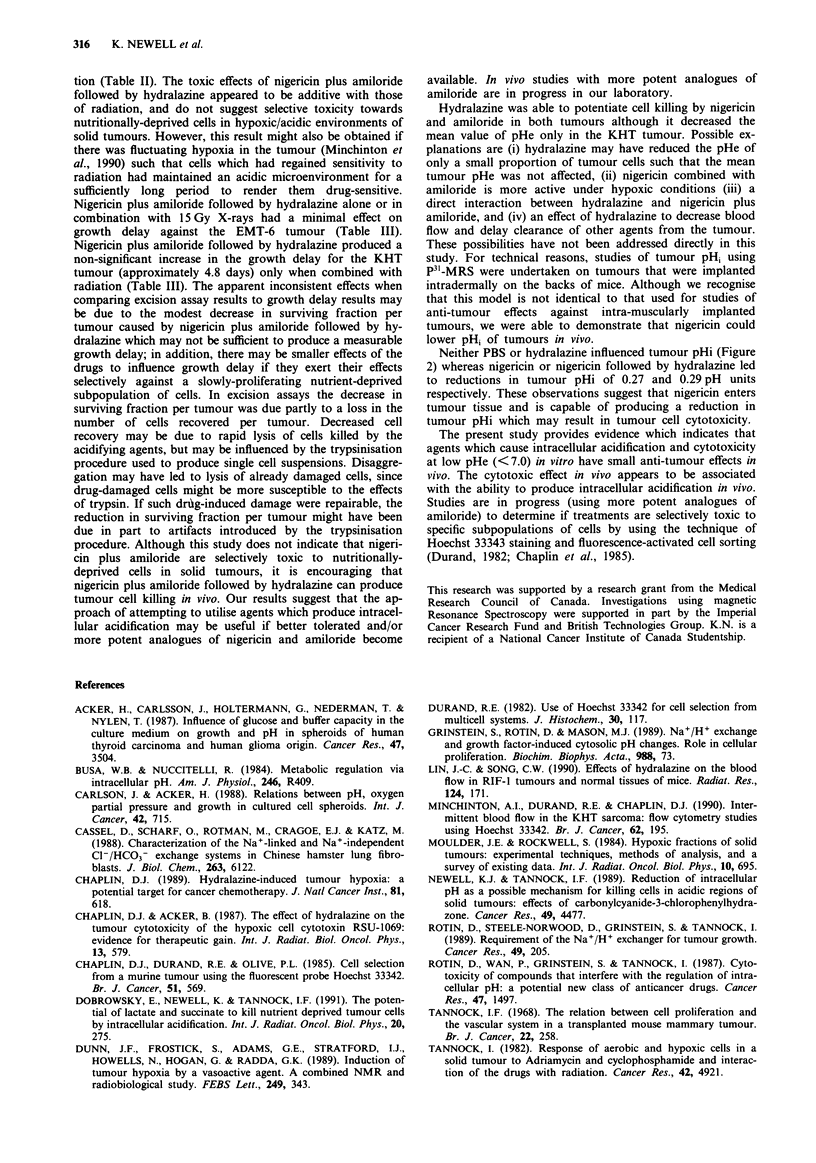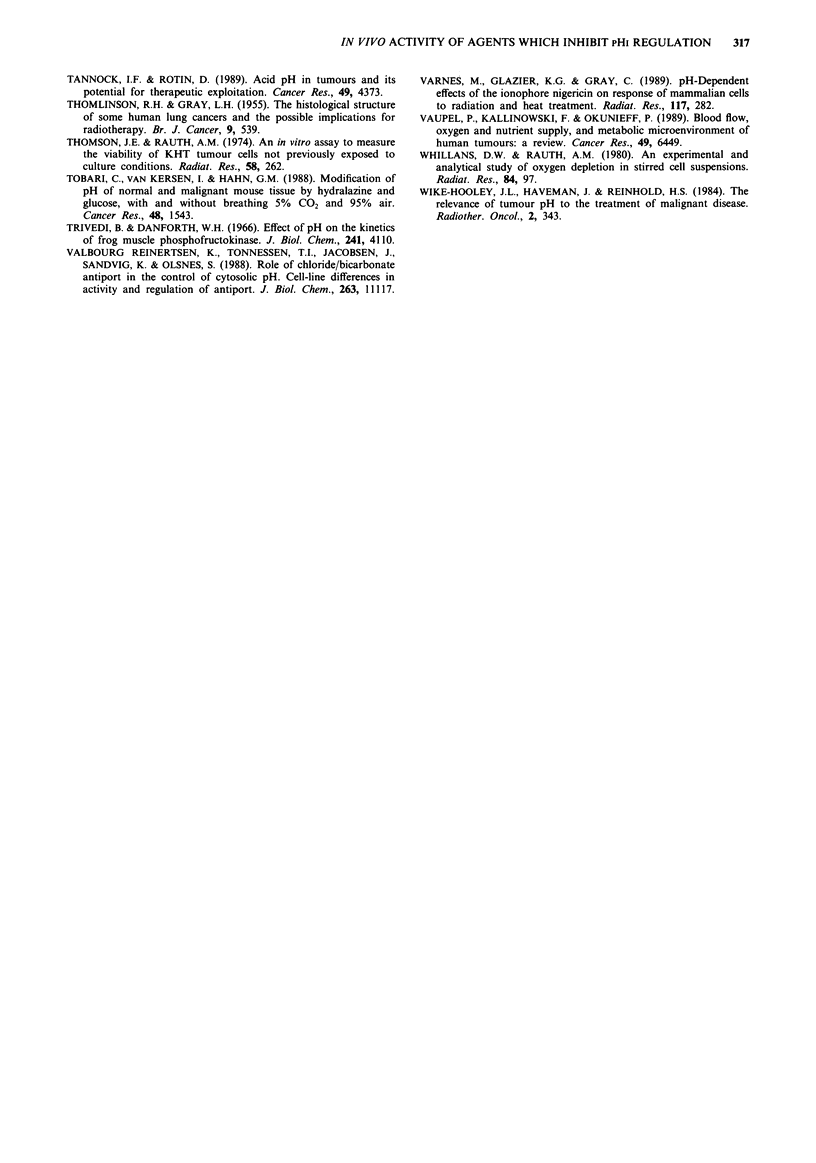# Effects of agents which inhibit the regulation of intracellular pH on murine solid tumours.

**DOI:** 10.1038/bjc.1992.262

**Published:** 1992-08

**Authors:** K. Newell, P. Wood, I. Stratford, I. Tannock

**Affiliations:** Department of Medical Biophysics, University of Toronto, Ontario, Canada.

## Abstract

Cell killing can be achieved in an acidic environment in tissue culture (medium pH less than 7.0) by agents (nigericin, carbonylcyanide-3-chlorophenylhydrazone (CCCP)) which transport protons from the extracellular space into the cytoplasm. Cell killing is enhanced when these agents are used in combination with compounds (amiloride, 4,4'-diisothiocyanostilbene-2,2'-disulfonic acid (DIDS)) which inhibit the membrane-based exchangers responsible for the regulation of intracellular pH (pHi). We describe experiments which assess the ability of these agents to kill tumour cells in spheroids and in vivo. Both nigericin and CCCP were observed to penetrate tissue based on their ability to kill tumour cells in spheroids. The mean extracellular pH (pHe) of the KHT fibrosarcoma and the EMT-6 sarcoma were observed to be 0.21 and 0.32 pH units more acidic than the mean pHe in muscle tissue. Intraperitoneal (i.p.) administration of the vasodilator hydralazine (10 mg kg-1) caused a reduction of the mean pHe of the KHT but not the EMT-6 tumour. Nigericin (2.5 mg kg-1, i.p.) plus amiloride (10 mg kg-1, i.p.) followed 30 min later by hydralazine (10 mg kg-1, i.p.) reduced the surviving fraction of cells in the KHT and EMT-6 tumours, but had minimal effects on growth delay. When KHT tumours were treated with 15 Gy X-rays followed immediately by nigericin plus amiloride and hydralazine a reduced surviving fraction as well as an increase in tumour growth delay was observed compared to radiation alone. The administration of nigericin (2.5 mg kg-1, i.p.) or the combination of nigericin (2.5 mg kg-1, i.p.) followed by hydralazine (10 mg kg-1, intravenous (i.v.)) resulted in reductions of tumour pHi of 0.27 and 0.29 pH units respectively as determined by 31P magnetic resonance spectroscopy (MRS). Our results show that the combination of nigericin and hydralazine (with or without amiloride) can kill cells in rodent solid tumours and that cell killing is associated with a reduction in the mean pHi of tumour cells.


Br. J. Cancer (1992), 66, 311–317 © Macmillan Press Ltd., 1992

### Effects of agents which inhibit the regulation of intracellular pH on murine solid tumours

K. Newell[1], P. Wood[2], I. Stratford[2] & I. Tannock[1]

[1]Department of Medical Biophysics, University of Toronto and Experimental Therapeutics Division, Ontario Cancer Institute, 500 Sherbourne Street, Toronto, Ontario M4X 1K9, Canada; [2]MRC Radiobiology Unit, Chilton, Didcot, Oxon OX11 0RD, UK.

**Summary** Cell killing can be achieved in an acidic environment in tissue culture (medium pH < 7.0) by agents (nigericin, carbonylcyanide-3-chlorophenylhydrazone (CCCP)) which transport protons from the extracellular space into the cytoplasm. Cell killing is enhanced when these agents are used in combination with compounds (amiloride, 4,4'-diisothiocyanostilbene-2,2'-disulfonic acid (DIDS)) which inhibit the membrane-based exchangers responsible for the regulation of intracellular pH (pHi). We describe experiments which assess the ability of these agents to kill tumour cells in spheroids and *in vivo*. Both nigericin and CCCP were observed to penetrate tissue based on their ability to kill tumour cells in spheroids. The mean extracellular pH (pHe) of the KHT fibrosarcoma and the EMT-6 sarcoma were observed to be 0.21 and 0.32 pH units more acidic than the mean pHe in muscle tissue. Intraperitoneal (i.p.) administration of the vasodilator hydralazine ($10\ mg\ kg^{-1}$) caused a reduction of the mean pHe of the KHT but not the EMT-6 tumour. Nigericin ($2.5\ mg\ kg^{-1}$, i.p.) plus amiloride ($10\ mg\ kg^{-1}$, i.p.) followed 30 min later by hydralazine ($10\ mg\ kg^{-1}$, i.p.) reduced the surviving fraction of cells in the KHT and EMT-6 tumours, but had minimal effects on growth delay. When KHT tumours were treated with 15 Gy X-rays followed immediately by nigericin plus amiloride and hydralazine a reduced surviving fraction as well as an increase in tumour growth delay was observed compared to radiation alone. The administraion of nigericin ($2.5\ mg\ kg^{-1}$, i.p.) or the combination of nigericin ($2.5\ mg\ kg^{-1}$, i.p.) followed by hydralazine ($10\ mg\ kg^{-1}$, intravenous (i.v.)) resulted in reductions of tumour pHi of 0.27 and 0.29 pH units respectively as determined by $^{31}P$ magnetic resonance spectroscopy (MRS). Our results show that the combination of nigericin and hydralazine (with or without amiloride) can kill cells in rodent solid tumours and that cell killing is associated with a reduction in the mean pHi of tumour cells.

The pH of solid tumours has been determined by using pH-sensitive microelectrodes which measure predominantly pHe, and by using $^{31}P$-MRS which measures primarily pHi (Wike-Hooley *et al.*, 1984; Vaupel *et al.*, 1989). Values of pHe in solid tumours extend over a broad range (median 6.9–7.0) and are on average approximately 0.5 pH units more acidic than normal tissue pHe (median 7.4–7.5) (Wike-Hooley *et al.*, 1984). Intracellular pH in solid tumours has been observed to have a wider range than that in normal tissue but median pHi (7.2) does not appear to be significantly lower in solid tumours as compared to normal tissue (Vaupel *et al.*, 1989). Taken together these results indicate that cells in solid tumours are surrounded by an acidic extracellular fluid and that tumour cells are actively regulating their pHi to physiological levels. Since the above techniques have poor spatial resolution, it seems probable that microenvironments exist within solid tumours which are more acidic than the observed median value of pHe.

As solid tumours enlarge, deficient vascularisation as compared to normal tissue usually results in poor delivery of oxygen to many regions within them (Thomlinson & Gray, 1955; Tannock, 1968). Cells in a hypoxic microenvironment are dependent on anaerobic glycolysis for energy production and consequently produce large amounts of lactic acid. Glycolytic activity resulting in lactic acid production is thought to be a cause of tumour acidity (Wike-Hooley *et al.*, 1984; Tannock & Rotin, 1989; Vaupel *et al.*, 1989). However, in recent experiments from this laboratory tumours were generated from glycolysis-deficient variant cells which developed an acidic microenvironment in the absence of lactate production (Newell *et al.*, unpublished observation). Regions of hypoxia probably coexist with regions of low pHe and possibly low pHi.

Hypoxic cells are known to be resistant to the effects of ionising radiation (Moulder & Rockwell, 1984; Vaupel *et al.*, 1989). Cells at a distance from the vasculature may be resistant to chemotherapy due to limited penetration of agents to these cells, or the low proliferative rate of nutritionally-deprived cells may render them insensitive to the effects of drugs active against cycling cells (e.g. Tannock, 1982; Chaplin *et al.*, 1985). Cells in a hypoxic/acidic microenvironment may therefore represent a subpopulation of tumour cells responsible for treatment failure. It might be possible to kill selectively cells in an acidic microenvironment within solid tumours by utilising the existing acidity to cause intracellular acidification.

Most cellular processes have pH optima at or near physiological pH (Trivedi & Danforth, 1966; Busa & Nuccitelli, 1984). The viability of cells in an acidic microenvironment depends therefore on the activity of membrane-based exchangers which regulate pHi (Rotin *et al.*, 1989). When cells are exposed to a low pHe environment, the $Na^+/H^+$ antiport (Grinstein *et al.*, 1989) and the $Na^+$-dependent $HCO_3^-/Cl^-$ exchanger (Cassel *et al.*, 1988; Valbourg-Reinertsen *et al.*, 1988) are the two major membrane-based exchangers involved in the regulation of pHi. The $Na^+/H^+$ antiport is inhibited by amiloride and the $Na^+$-dependent $HCO_3^-/Cl^-$ exchanger is inhibited by stilbene derivatives such as DIDS.

Our laboratory has shown that it is possible to kill selectively cells in an acidic environment *in vitro* by using agents which cause intracellular acidification. Intracellular acidification leading to cell death was accomplished *in vitro* using agents which transport $H^+$ from the extracellular space into the cytoplasm and this effect was enhanced by agents which inhibit the membrane-based mechanisms which regulate pHi. The ionophore nigericin (Rotin *et al.*, 1987), which is capable of exchanging intracellular $K^+$ for extracellular $H^+$, or CCCP (Newell & Tannock, 1989), which is capable of transporting $H^+$ equivalents into cells, as well as the weak acid succinate (Dobrowsky *et al.*, 1991), were cytotoxic to tumour cells *in vitro* only at pHe < 6.5. When nigericin or CCCP was combined with amiloride and/or the stilbene derivative DIDS, agents which inhibit the membrane-based exchangers responsible for the regulation of pHi, cytotoxicity was observed at pHe < 7.0. In these *in vitro* studies cytotoxicity was

Correspondence: I. Tannock.
Received 10 December 1991; and in revised form 11 March 1992.

associated with the ability to produce intracellular acidification.

The objective of the present study was to determine if agents which produce intracellular acidification and are selectively toxic at low pHe *in vitro* have anti-tumour effects against two murine transplantable tumours.

### Materials and methods

### *Cells*

At the Ontario Cancer Institute, the KHT fibrosarcoma and the EMT-6 murine sarcoma cells were maintained *in vitro* in α-minimum essential medium (α-MEM) supplemented with 10% foetal bovine serum (FBS) and 0.1 mg ml$^{-1}$ kanamycin (complete medium). Both cell lines were maintained in culture for eight passages and then were passaged in syngeneic hosts. Routine tests for *mycoplasma* revealed that the cell lines were free from contamination. Tumour cells were discarded at 4–6 months intervals and re-initiated from frozen stock.

### *Spheroids*

Spheroids were used in some experiments as a model of intermediate complexity between single cells and solid tumours to assess the ability of our agents to penetrate into tissue. Spheroids were grown from EMT-6 murine sarcoma cells. EMT-6 spheroids were initiated by seeding $10^4$ cells ml$^{-1}$ in 15 ml of complete medium in a non-tissue culture treated plastic dish. After 4 days cell aggregates were transferred into spinner flasks containing 200 ml of α-MEM minus bicarbonate supplemented with 25 mM hydroxyethylpiperazine-N'-2-ethanesulfonic acid (Hepes) (pH 7.4), 10% FBS and 0.1 mg ml$^{-1}$ kanamycin (spheroid medium). Medium was changed every 48 h. Spheroid diameter was determined by measuring orthogonal diameters of a minimum of 30 spheroids and then determining a geometric mean diameter for the growing population. Spheroids were used for experiments when they had attained a mean diameter of approximately 800 μm.

To assess cell survival following exposure to potentially toxic agents, spheroids were washed once with α-MEM plus 10% FBS buffered with bicarbonate/2[N-morpholino]-ethanesulfonic acid (Mes) (25 mM) to the desired pH and placed in 50 ml spinner flasks containing the same medium (Newell & Tannock, 1989). Spinner flasks were placed in a 37°C water bath and humidified 5% $CO_2$/95% air was continuously flowed over the suspensions (Whillans & Rauth, 1980). Medium pH drifted a maximum of 0.1 pH units during the exposure period. After 30 min equilibration, drugs were added. Samples were taken and spheroids were washed once with fresh complete medium and disaggregated by trypsinisation (0.05% trypsin in 0.53 mM ethylenediaminetetraacetic acid (Gibco; Grand Island, New York)) for 20 min at 37°C. The resulting single cell suspensions were counted electronically, diluted and plated in triplicate. After 11 days plates were stained and colonies containing greater than 50 cells were counted.

### *Tumours*

At the Ontario Cancer Institute, the KHT fibrosarcoma and the EMT-6 sarcoma were propagated in 8–12 week old inbred female C3H/HeJ and BALB/c BYJ mice respectively. Mice were purchased from Jackson Laboratories (Bar Harbour, Maine). Tumours were initiated by intramuscular injection of 2.5–5.0 × $10^5$ cells into the left hind leg. Growth of tumours was monitored by passing the tumour-bearing leg through a strip of lucite with graded size holes. The diameter of the tumour-bearing leg was converted to an estimate of tumour weight using a previously defined calibration curve. Tumours were used for experiments when they had attained 0.3–0.5 g in weight, which required approximately 9 days for both tumour types.

The subline of the KHT fibrosarcoma that was used for studies of magnetic resonance spectroscopy (MRS) at the MRC Unit, UK was maintained by i.m. *in vivo* passage in C3H mice, with return to frozen stock after 15–20 passages. For MRS experiments tumours were generated by injecting a single cell suspension containing approximately 2 × $10^5$ cells intradermally on the back of the mouse. Tumours were studied after approximately 10 days growth, when they had a diameter of 6–8 mm.

### *Measurement of tumour pHe*

Mice were anaesthetised with tribomoethanol 0.50 mg kg$^{-1}$ mouse weight (Avertin). Measurements of pHe were made using a miniature glass electrode (model MI-408B, Microelectrodes Inc.) against a silver-silver chloride reference electrode (model MI-402, Microelectrodes Inc.) using a portable pH meter (model pH 103, Corning). The reference electrode was inserted subcutaneously on the back and the pH electrode was inserted directly into the tumour or muscle after incising the overlying skin. Measurements of tumour pHe were made at increments of 50–75 μm along a single track at a depth of 300–500 μm into the tumour by using a specially constructed micrometer to advance the electrode. A mean of 6 and a minimum of 4 pHe measurements were made per tumour.

### *Drugs*

CCCP, nigericin, amiloride, hydralazine and all other chemicals were purchased from Sigma (St. Louis, Missouri). Mice were injected with various combinations of CCCP dissolved in 10% ethanol, nigericin dissolved in 10% ethanol, amiloride dissolved in sterile water, and hydralazine dissolved in sterile water. Nigericin was prepared and stored in glass vials due to its known ability to bind to plastic (Varnes *et al.*, 1989). All drugs were delivered i.p. in a volume of 0.01 mg g$^{-1}$ body weight except for $^{31}$P-MRS experiments when hydralazine was administered i.v. Radiation was delivered to the tumour-bearing limb of unanaesthetised mice restrained in a specially designed jig. The radiation source was a double-headed 100 kVp X-ray unit which has a dose rate of 10.2 Gy min$^{-1}$.

### *Excision assay*

Tumours were excised 20–24 h after treatment, weighed and then minced with scissors in phosphate buffered saline (PBS). Single cell suspensions were prepared by enzymatic digestion with trypsin (Difco) and DNAse I (Sigma) (Thomson & Rauth, 1974). Tumour cell suspensions were stained with trypan blue and dye-excluding cells were counted with a haemocytometer. Tumour cell suspensions were then diluted and plated in triplicate in complete medium. Plates were stained 11 days later and colonies containing greater than 50 cells were counted. Surviving fraction per tumour was calculated according to: SF/tumour = (plating efficiency treated/plating efficiency control) × ((cells/gram treated)/(cells/gram control)).

### *Growth delay assay*

Mice bearing tumours in the range of 0.3–0.5 g (8.5–9.5 mm leg diameter) were identified with ear tags and randomly distributed into treatment groups (minimum five mice per group). Mice were treated and leg diameters were recorded to the nearest 0.5 mm every 2–3 days by an observer who was unaware of the treatment history. Measurements were converted to estimates of tumour weight, and growth curves were compared for treated and control tumours. Mice were killed humanely when tumours attained a weight of approximately 1.5 g.

*Tumour $^{31}P$-MRS measurements*

Unanaesthetised mice bearing intradermal KHT tumours were restrained in a specially designed jig. MRS experiments were performed using a 4.7 Tesla 30 cm horizontal bore magnet (Oxford Instruments), interfaced with a SISCO 200 spectrometer. A 7 mm surface coil was placed over the tumour for Rf transmission and signal collection. Acquisition parameters were set to minimise contamination from underlying tissue. Each spectrum consisted of 256 scans with a 2 s delay time, giving a total collection time of approximately 7 min per spectrum. Spectra from a single undistributed mouse were recorded at 15 min intervals up to 120 min after drug administration. Values of pHi were calculated by examining the change in the chemical shift of the inorganic phosphate peak relative to α-ATP and γ-ATP peaks. Since phosphocreatine was not present in the spectra of every tumour, this peak was not used to calculate pHi.

### Results

*Spheroid toxicity*

EMT-6 spheroids were exposed to concentrations of CCCP (15μM) and nigericin (3.4μM) which had been shown previously to be toxic to single cell suspensions (Rotin *et al.*, 1987; Newell & Tannock, 1989). Cell killing was not observed for spheroids exposed to CCCP or nigericin at pH 7.4, or spheroids exposed to low pHe (6.4) in the absence of ionophores (Figure 1). A time dependent decrease in surviving fraction was observed for spheroids exposed to CCCP or nigericin at pHe 6.4 (Figure 1), indicating that both drugs were able to penetrate to the interior cells of spheroids.

*Tumour pHe*

The mean values of pHe in KHT (6.84) and EMT-6 (6.75) tumours were observed to be significantly more acidic than the mean pHe of muscle (7.06) (Table I). Since the *in vitro* cytotoxicity of the compounds to be tested increased sharply at lower values of pHe, we attempted to decrease selectively tumour pHe by the i.p. administration of the vasodilator hydralazine, which has been shown to cause large transient increases in the hypoxic fraction of rodent solid tumours (Chaplin & Acker, 1987; Chaplin, 1989; Dunn *et al.*, 1989). Forty-five minutes after hydralazine treatment there was a small increase in the pHe differential between tumour and muscle tissue for both tumour types (Table I). For KHT tumours, tumour pHe was reduced and muscle pHe remained the same, whereas for EMT-6 tumours, tumour pHe remained the same and muscle pHe was increased.

*Effect of nigericin and hydralazine on tumour pHi*

No reduction in pHi in KHT tumours was observed in control mice injected with PBS (Figure 2) or with hydralazine (10 mg kg$^{-1}$, i.v.) (data not shown). Nigericin (2.5 mg kg$^{-1}$, i.p.) caused a significant reduction in pHi with a maximum decrease of 0.27 pH units occurring 45 min after injection (Figure 2). The combination of nigericin followed by hydralazine was observed to cause a similar decrease in pHi (maximum 0.29 pH units) with a trend to a more prolonged period of acidification (Figure 2).

*CCCP*

The dose of CCCP which could be administered to mice was not limited by toxicity but by solubility of CCCP in sterile

**Figure 1** Surviving fraction of cells from EMT-6 spheroids. Spheroids were exposed to the following conditions pH 7.4, 6.4 (data not shown), pH 7.4, 6.4 plus 15 μM CCCP (□,■), and pH 7.4, 6.4 plus 3.4 μM nigericin (△,▲). No decrease in surviving fraction was observed for spheroids exposed to pH 7.4 or pH 6.4 in the absence of ionophores. Points represent mean and range from one experiment (range of values less than vertical extent of symbols). Qualitatively similar results have been obtained in a repeat experiment.

**Figure 2** The effect of PBS (○), nigericin (2.5 mg kg$^{-1}$) (□), and nigericin (2.5 mg kg$^{-1}$) and hydralazine (10 mg kg$^{-1}$) (△) on pHi in KHT tumours as determined by $^{31}$P-NMR. Points and error bars represent mean ± standard error from at least six tumours.

**Table I** Effect of hydralazine on tumour pHe

| Tissue | pHe KHT | pHe EMT-6 | pHe 45 min after hydralazine (10 mg kg$^{-1}$) KHT | pHe 45 min after hydralazine (10 mg kg$^{-1}$) EMT-6 |
|---|---|---|---|---|
| Muscle | 7.05 (0.08)[a] | 7.07 (0.08) | 7.05 (0.08) | 7.21 (0.04) |
| Tumour | 6.84 (0.06) | 6.75 (0.06) | 6.64 (0.11) | 6.82 (0.08) |
| ΔpHe | 0.21 (0.10)[b] | 0.32 (0.10) | 0.41 (0.14) | 0.39 (0.09) |

[a]Values represent mean ± s.e.mean from at least six tumours. [b]ΔpHe = (mean muscle pHe-mean tumour pHe).

water containing 10% ethanol. A dose of $1.0\,g\,kg^{-1}$ CCCP was well tolerated alone or in combination with $10\,mg\,kg^{-1}$ amiloride and/or $10\,mg\,kg^{-1}$ hydralazine. Similar results were obtained when CCCP was dissolved in alternative solvents such as dimethylsulfoxide and N-N-dimethylacetamide.

Treatment of KHT tumours with CCCP plus amiloride followed 30 min later by hydralazine alone or in combination with 15 Gy X-rays did not lead to a decrease in surviving fraction per KHT tumour in excision assays (data not shown). No growth delay was observed for mice treated with CCCP plus amiloride followed by hydralazine either when these three agents were given alone, or in combination with 15 Gy X-rays (data not shown).

*Nigericin*

The maximum tolerated dose of nigericin dissolved in sterile water containing 10% ethanol was approximately $4.0\,mg\,kg^{-1}$ alone and approximately $2.5\,mg\,kg^{-1}$ in combination with amiloride ($10\,mg\,kg^{-1}$) and/or hydralazine ($10\,mg\,kg^{-1}$). Maximum tolerated dose was defined as the dose of drug which did not cause animal death within 30 days.

After excision of tumours the number of dye-excluding tumour cells per gram of tumour tissue was determined. It was observed that only nigericin followed by hydralazine or nigericin plus amiloride followed by hydralazine caused significant decreases in the number of dye-excluding cells per gram of tumour recovered (Table II). A similar reduction in cell recovery was observed when drugs were combined with 15 Gy X-rays (Table II). These conditions also led to a decrease in surviving fraction per tumour (Table II). The decrease in surviving fraction per tumour caused by the drug combination appeared to be additive to the decrease in surviving fraction per tumour caused by radiation. Similar effects were observed for the EMT-6 tumour (data not shown).

The decrease in surviving fraction per tumour for nigericin followed by hydralazine was observed to be dependent on the dose of nigericin (Figure 3a) and on the dose of hydralazine (Figure 3b). A decrease in surviving fraction per tumour was observed when hydralazine was administered 3 h prior to or 3 h after nigericin plus amiloride (Figure 4). Maximum reduction in surviving fraction per tumour was observed when hydralazine was administered from 1 h before to 1 h after nigericin.

The effects of nigericin plus amiloride followed by hydralazine on tumour growth delay are presented in Figure 5. When used alone these agents did not lead to a significant delay in tumour growth. When used with radiation, no additional growth delay was observed for EMT-6 tumours, whereas for the KHT tumour there was a trend to increased growth delay when drugs were used in combination with radiation (mean 12.3 days) as compared to radiation alone (mean 7.5 days) (Table III).

### Discussion

The results from this study indicate that the ionophore nigericin, which produces pHe-dependent cytotoxicity via intracellular acidification *in vitro*, is capable of killing cells in murine solid tumours.

Microelectrode measurements of pHe within spheroids have shown that pHe decreases as distance from the spheroid surface increases (Acker *et al.*, 1987; Carlson & Acker, 1988). In medium at pH 7.4, minimum pHe at a depth of 200 μm

**Figure 3** a, The effect of the dose of nigericin on surviving fraction per KHT tumour for mice treated with the indicated dose of nigericin followed 30 min later by hydralazine ($10\,mg\,kg^{-1}$). b, The effect of the dose of hydralazine on the surviving fraction per KHT tumour. Mice were treated with nigericin ($2.5\,mg\,kg^{-1}$) followed 30 min later by the indicated dose of hydralazine. Points and error bars represent mean and range from at least two experiments.

**Table II** Number of dye excluding cells ($\times 10^{-7}$) recovered per gram of tumour and surviving fraction per tumour for the KHT tumour

| Treatment | *Drugs alone* # *cells per gram* | *S.F. per tumour* | *Drugs plus 15 Gy X-rays* # *cells per gram* | *S.F. per tumour* |
|---|---|---|---|---|
| Control | 7.7 (6.9–12.0) | 1.0 (0.85–1.0) | 4.8 (2.5–8.2) | $2.6 \times 10^{-2}$ ($2.1–3.0 \times 10^{-2}$) |
| Nigericin + Amiloride | 7.3 (4.0–10.1) | 0.73 (0.82–0.64) | 6.2 (2.0–10.4) | $2.7 \times 10^{-2}$ ($2.5–2.8 \times 10^{-2}$) |
| Hydralazine | 9.0 (7.9–10.1) | 0.70 (0.62–0.82) | 7.7 (6.4–9.0) | $1.2 \times 10^{-2}$ ($0.9–2.5 \times 10^{-2}$) |
| Nigericin → Hydralazine | 0.7 (0.5–1.0) | $1.3 \times 10^{-2}$ ($0.8–1.8 \times 10^{-2}$) | – | – |
| Nigericin + Amiloride → Hydralazine | 1.5 (0.7–2.2) | $3.7 \times 10^{-2}$ ($3.5–3.8 \times 10^{-2}$) | 1.4 (0.9–1.9) | $3.4 \times 10^{-4}$ ($2.8–3.6 \times 10^{-4}$) |

Values represent mean (range) from at least two experiments.

**Figure 4** The effect of the time of hydralazine administration on the surviving fraction per KHT tumour. Mice were treated with nigericin (2.5 mg $kg^{-1}$) plus amiloride (10 mg $kg^{-1}$) and hydralazine (10 mg $kg^{-1}$), was given at the indicated time relative to the other agents. Points and error bars represent mean and range from at least two experiments.

**Figure 5** Growth curves for EMT-6 **a**, and KHT **b**, tumours. Mice were treated with control (○), nigericin (2.5 mg $kg^{-1}$) and amiloride (10 mg $kg^{-1}$) followed 30 min later by hydralazine (10 mg $kg^{-1}$) (●), 15 Gy X-rays (□), and 15 Gy X-rays plus drugs (■). Points and error bars represent mean ± s.e. for at least five tumours.

from the surface of spheroids has been observed to be approximately 6.8. Cell killing was not observed when EMT-6 spheroids were exposed to CCCP or nigericin at pHe 7.4. Since CCCP and nigericin have been shown previously to be selectively toxic at pHe's less than 6.5, the failure to observe cell killing at pHe 7.4 was probably due to the fact that at most a small proportion of cells within the spheroids had ambient pHe within the range necessary for cytotoxicity of CCCP or nigericin. When spheroids were incubated in medium at low pHe and exposed to CCCP or nigericin, time dependent cytotoxicity was observed with several orders of magnitude of cell killing (Figure 1). This result demonstrates that CCCP and nigericin are capable of penetrating into tissue and killing cells.

The mean pHe in KHT (6.84) and EMT-6 (6.75) tumours growing in the legs of mice was observed to be 0.21 and 0.32 pH units more acidic than muscle pHe (Table III). The observed values of pHe for KHT and EMT-6 tumours are consistent with those reported for other rodent solid tumours (Wike-Hooley *et al.*, 1984); however, mean values of pHe within KHT and EMT-6 tumours were above the threshold (pHe 6.5) for cytotoxicity by nigericin or CCCP alone (Rotin *et al.*, 1987; Newell & Tannock, 1989). Nigericin and CCCP were therefore combined with the $Na^+/H^+$ exchange inhibitor amiloride which has been shown to allow killing of cells by CCCP and nigericin at values of pHe below 7.0.

We could not demonstrate *in vivo* toxicity of CCCP when used in high concentration (1.0 g $kg^{-1}$ body weight) either alone or in combination with other agents. The lack of cytotoxicity for CCCP may have been due to the limited solubility of CCCP in aqueous solutions which may prevent CCCP from reaching toxic concentrations within tumours. We have not performed pharmacological studies in an attempt to address directly this possibility.

Nigericin combined with amiloride also was not cytotoxic to KHT or EMT-6 tumours in excision assays or in growth delay experiments. Failure to observe *in vivo* toxicity may have been due to pHe values that were too high to allow cell killing at the doses achieved. We therefore used the vasodilator hydralazine in an attempt to reduce tumour pHe. Hydralazine has been shown previously to produce a dose-dependent reduction in tumour blood flow (Chaplin, 1989; Lin & Song, 1990). This decrease in tumour blood flow may lead to a decrease in tumour pHe because the rate of glycolysis may increase under induced anaerobic conditions, leading to accumulation of lactic acid, and because of reduced clearance of this and other metabolic acids. Hydralazine caused a decrease in pHe of the KHT tumour with little change in pHe of EMT-6 tumours. Our results with the EMT-6 tumour are consistent with a previous study which reported that hydralazine did not reduce pHe in RIF-1 tumours (Tobari *et al.*, 1988).

When nigericin plus amiloride was combined with hydralazine a decrease in surviving fraction was observed when the agents were given alone or in combination with 15 Gy radia-

**Table III** Delay in growth of KHT and EMT-6 tumours following various treatments

| | *KHT* | | *EMT-6* | |
|---|---|---|---|---|
| *Treatment* | *Days to 1 g* | *Growth delay* | *Days to 1 g* | *Growth delay* |
| Control | 4.7 (1.0) | – | 5.5 (0.9) | – |
| Nigericin + Amiloride→ Hydralazine | 5.3 (0.5) | 0.6 | 7.6 (1.3) | 2.1 |
| 15 Gy X-rays | 12.2 (1.5) | 7.5 | 16.5 (1.4) | 11.0 |
| 15 Gy X-rays→ Nigericin + Amiloride→ Hydralazine | 17.0 (4.6) | 12.3 | 16.2 (1.3) | 10.7 |

The values for days to 1 g represent mean ± s.e. from two growth delay experiments. Growth delay = [(days to 1 $g)_{treated}$-(days to 1 $g)_{control}$].

tion (Table II). The toxic effects of nigericin plus amiloride followed by hydralazine appeared to be additive with those of radiation, and do not suggest selective toxicity towards nutritionally-deprived cells in hypoxic/acidic environments of solid tumours. However, this result might also be obtained if there was fluctuating hypoxia in the tumour (Minchinton *et al.*, 1990) such that cells which had regained sensitivity to radiation had maintained an acidic microenvironment for a sufficiently long period to render them drug-sensitive. Nigericin plus amiloride followed by hydralazine alone or in combination with 15 Gy X-rays had a minimal effect on growth delay against the EMT-6 tumour (Table III). Nigericin plus amiloride followed by hydralazine produced a non-significant increase in the growth delay for the KHT tumour (approximately 4.8 days) only when combined with radiation (Table III). The apparent inconsistent effects when comparing excision assay results to growth delay results may be due to the modest decrease in surviving fraction per tumour caused by nigericin plus amiloride followed by hydralazine which may not be sufficient to produce a measurable growth delay; in addition, there may be smaller effects of the drugs to influence growth delay if they exert their effects selectively against a slowly-proliferating nutrient-deprived subpopulation of cells. In excision assays the decrease in surviving fraction per tumour was due partly to a loss in the number of cells recovered per tumour. Decreased cell recovery may be due to rapid lysis of cells killed by the acidifying agents, but may be influenced by the trypsinisation procedure used to produce single cell suspensions. Disaggregation may have led to lysis of already damaged cells, since drug-damaged cells might be more susceptible to the effects of trypsin. If such drug-induced damage were repairable, the reduction in surviving fraction per tumour might have been due in part to artifacts introduced by the trypsinisation procedure. Although this study does not indicate that nigericin plus amiloride are selectively toxic to nutritionally-deprived cells in solid tumours, it is encouraging that nigericin plus amiloride followed by hydralazine can produce tumour cell killing *in vivo*. Our results suggest that the approach of attempting to utilise agents which produce intracellular acidification may be useful if better tolerated and/or more potent analogues of nigericin and amiloride become available. *In vivo* studies with more potent analogues of amiloride are in progress in our laboratory.

Hydralazine was able to potentiate cell killing by nigericin and amiloride in both tumours although it decreased the mean value of pHe only in the KHT tumour. Possible explanations are (i) hydralazine may have reduced the pHe of only a small proportion of tumour cells such that the mean tumour pHe was not affected, (ii) nigericin combined with amiloride is more active under hypoxic conditions (iii) a direct interaction between hydralazine and nigericin plus amiloride, and (iv) an effect of hydralazine to decrease blood flow and delay clearance of other agents from the tumour. These possibilities have not been addressed directly in this study. For technical reasons, studies of tumour $pH_i$ using $P^{31}$-MRS were undertaken on tumours that were implanted intradermally on the backs of mice. Although we recognise that this model is not identical to that used for studies of anti-tumour effects against intra-muscularly implanted tumours, we were able to demonstrate that nigericin could lower $pH_i$ of tumours *in vivo*.

Neither PBS or hydralazine influenced tumour pHi (Figure 2) whereas nigericin or nigericin followed by hydralazine led to reductions in tumour pHi of 0.27 and 0.29 pH units respectively. These observations suggest that nigericin enters tumour tissue and is capable of producing a reduction in tumour pHi which may result in tumour cell cytotoxicity.

The present study provides evidence which indicates that agents which cause intracellular acidification and cytotoxicity at low pHe ($<7.0$) *in vitro* have small anti-tumour effects *in vivo*. The cytotoxic effect *in vivo* appears to be associated with the ability to produce intracellular acidification *in vivo*. Studies are in progress (using more potent analogues of amiloride) to determine if treatments are selectively toxic to specific subpopulations of cells by using the technique of Hoechst 33343 staining and fluorescence-activated cell sorting (Durand, 1982; Chaplin *et al.*, 1985).

This research was supported by a research grant from the Medical Research Council of Canada. Investigations using magnetic Resonance Spectroscopy were supported in part by the Imperial Cancer Research Fund and British Technologies Group. K.N. is a recipient of a National Cancer Institute of Canada Studentship.

### References

ACKER, H., CARLSSON, J., HOLTERMANN, G., NEDERMAN, T. & NYLEN, T. (1987). Influence of glucose and buffer capacity in the culture medium on growth and pH in spheroids of human thyroid carcinoma and human glioma origin. *Cancer Res.*, **47**, 3504.

BUSA, W.B. & NUCCITELLI, R. (1984). Metabolic regulation via intracellular pH. *Am. J. Physiol.*, **246**, R409.

CARLSON, J. & ACKER, H. (1988). Relations between pH, oxygen partial pressure and growth in cultured cell spheroids. *Int. J. Cancer*, **42**, 715.

CASSEL, D., SCHARF, O., ROTMAN, M., CRAGOE, E.J. & KATZ, M. (1988). Characterization of the $Na^+$-linked and $Na^+$-independent $Cl^-/HCO_3^-$ exchange systems in Chinese hamster lung fibroblasts. *J. Biol. Chem.*, **263**, 6122.

CHAPLIN, D.J. (1989). Hydralazine-induced tumour hypoxia: a potential target for cancer chemotherapy. *J. Natl Cancer Inst.*, **81**, 618.

CHAPLIN, D.J. & ACKER, B. (1987). The effect of hydralazine on the tumour cytotoxicity of the hypoxic cell cytotoxin RSU-1069: evidence for therapeutic gain. *Int. J. Radiat. Biol. Oncol. Phys.*, **13**, 579.

CHAPLIN, D.J., DURAND, R.E. & OLIVE, P.L. (1985). Cell selection from a murine tumour using the fluorescent probe Hoechst 33342. *Br. J. Cancer*, **51**, 569.

DOBROWSKY, E., NEWELL, K. & TANNOCK, I.F. (1991). The potential of lactate and succinate to kill nutrient deprived tumour cells by intracellular acidification. *Int. J. Radiat. Oncol. Biol. Phys.*, **20**, 275.

DUNN, J.F., FROSTICK, S., ADAMS, G.E., STRATFORD, I.J., HOWELLS, N., HOGAN, G. & RADDA, G.K. (1989). Induction of tumour hypoxia by a vasoactive agent. A combined NMR and radiobiological study. *FEBS Lett.*, **249**, 343.

DURAND, R.E. (1982). Use of Hoechst 33342 for cell selection from multicell systems. *J. Histochem.*, **30**, 117.

GRINSTEIN, S., ROTIN, D. & MASON, M.J. (1989). $Na^+/H^+$ exchange and growth factor-induced cytosolic pH changes. Role in cellular proliferation. *Biochim. Biophys. Acta.*, **988**, 73.

LIN, J.-C. & SONG, C.W. (1990). Effects of hydralazine on the blood flow in RIF-1 tumours and normal tissues of mice. *Radiat. Res.*, **124**, 171.

MINCHINTON, A.I., DURAND, R.E. & CHAPLIN, D.J. (1990). Intermittent blood flow in the KHT sarcoma: flow cytometry studies using Hoechst 33342. *Br. J. Cancer*, **62**, 195.

MOULDER, J.E. & ROCKWELL, S. (1984). Hypoxic fractions of solid tumours: experimental techniques, methods of analysis, and a survey of existing data. *Int. J. Radiat. Oncol. Biol. Phys.*, **10**, 695.

NEWELL, K.J. & TANNOCK, I.F. (1989). Reduction of intracellular pH as a possible mechanism for killing cells in acidic regions of solid tumours: effects of carbonylcyanide-3-chlorophenylhydrazone. *Cancer Res.*, **49**, 4477.

ROTIN, D., STEELE-NORWOOD, D., GRINSTEIN, S. & TANNOCK, I. (1989). Requirement of the $Na^+/H^+$ exchanger for tumour growth. *Cancer Res.*, **49**, 205.

ROTIN, D., WAN, P., GRINSTEIN, S. & TANNOCK, I. (1987). Cytotoxicity of compounds that interfere with the regulation of intracellular pH: a potential new class of anticancer drugs. *Cancer Res.*, **47**, 1497.

TANNOCK, I.F. (1968). The relation between cell proliferation and the vascular system in a transplanted mouse mammary tumour. *Br. J. Cancer*, **22**, 258.

TANNOCK, I. (1982). Response of aerobic and hypoxic cells in a solid tumour to Adriamycin and cyclophosphamide and interaction of the drugs with radiation. *Cancer Res.*, **42**, 4921.

TANNOCK, I.F. & ROTIN, D. (1989). Acid pH in tumours and its potential for therapeutic exploitation. *Cancer Res.*, **49**, 4373.

THOMLINSON, R.H. & GRAY, L.H. (1955). The histological structure of some human lung cancers and the possible implications for radiotherapy. *Br. J. Cancer*, **9**, 539.

THOMSON, J.E. & RAUTH, A.M. (1974). An *in vitro* assay to measure the viability of KHT tumour cells not previously exposed to culture conditions. *Radiat. Res.*, **58**, 262.

TOBARI, C., VAN KERSEN, I. & HAHN, G.M. (1988). Modification of pH of normal and malignant mouse tissue by hydralazine and glucose, with and without breathing 5% $CO_2$ and 95% air. *Cancer Res.*, **48**, 1543.

TRIVEDI, B. & DANFORTH, W.H. (1966). Effect of pH on the kinetics of frog muscle phosphofructokinase. *J. Biol. Chem.*, **241**, 4110.

VALBOURG REINERTSEN, K., TONNESSEN, T.I., JACOBSEN, J., SANDVIG, K. & OLSNES, S. (1988). Role of chloride/bicarbonate antiport in the control of cytosolic pH. Cell-line differences in activity and regulation of antiport. *J. Biol. Chem.*, **263**, 11117.

VARNES, M., GLAZIER, K.G. & GRAY, C. (1989). pH-Dependent effects of the ionophore nigericin on response of mammalian cells to radiation and heat treatment. *Radiat. Res.*, **117**, 282.

VAUPEL, P., KALLINOWSKI, F. & OKUNIEFF, P. (1989). Blood flow, oxygen and nutrient supply, and metabolic microenvironment of human tumours: a review. *Cancer Res.*, **49**, 6449.

WHILLANS, D.W. & RAUTH, A.M. (1980). An experimental and analytical study of oxygen depletion in stirred cell suspensions. *Radiat. Res.*, **84**, 97.

WIKE-HOOLEY, J.L., HAVEMAN, J. & REINHOLD, H.S. (1984). The relevance of tumour pH to the treatment of malignant disease. *Radiother. Oncol.*, **2**, 343.